# The Chelsea Hospital for Women

**Published:** 1894-07-28

**Authors:** 


### The Hospital

AN INSTITUTIONAL JOURNAL OF

The Medical Sciences and Hospital Administration,

WITH

AN EXTRA NURSING SUPPLEMENT.

VOL. XVI.—No. 409.] [JULY 28, 1894.

### THE CHELSEA HOSPITAL FOR WOMEN.

THE report just presented to Earl Cadogan, President of the Chelsea Hospital for Women, by the Special Committee appointed to inquire into the mortality which has attended the proceedings of that institution, is a document requiring the most anxious consideration from all who are concerned in hospital management, or who desire that the work of a hospital should combine the maximum of good with the minimum of evil. We publish a full abstract of this report in another column. It is hardly necessary to say that aggregation of the sick is not in itself desirable, or that it is tolerated only for the sake of economy, and because it permits the application of the highest medical skill to a larger number of persons than could otherwise be dealt with; while the attendant disadvantages may be rendered comparatively trivial by the exercise of unremitting care.

It will be remembered that in February last Dr. Parkes, the Medical Officer of Health for Chelsea, felt it his duty to report to his Vestry that the mortality in the hospital was excessive, and that its sanitary state was unsatisfactory. As one con sequence of his action the building was closed for a time; and his report obtained wide publicity through the columns of the non-medical press. The president of the hospital, in consequence of these circumstances, nominated a committee to inquire into the facts. Of this committee Lord Balfour of Burleigh was chairman, while Sir John Williams and Mr. Howse were representatives of the Royal Colleges of Physicians and Surgeons respectively.

The report of the committee was unanimous. It begins by a simple narrative of the events which led up to the inquiry, and by an expression of regret that one passage in Dr. Parkes' official letter to the Vestry should have been so worded as to lead to misunderstanding. Dr. Parkes wrote, referring to certain operations which were intended to mitigate pain and discomfort, but not primarily to save life, "that the question of the justifiability of such operations must arise, unless it is possible to reduce the risk of fatal issue from them to an extremely low figure." In his mind the whole of the sentence was governed by the words subsequent to "unless," and, when so governed, it is little more than a truism. Some readers of the report, however, failed to notice, or chose to ignore, the concluding member of the sentence, and assumed that Dr. Parkes had challenged the propriety of the operations in any case. This, according to his own subsequent statement, he neither did nor intended to do.

The general result brought out by the committee, after an inquiry which seems to have been as complete as circumstances would allow, was that the mortality in the hospital after ovariotomy, during the year 1893, was six deaths out of thirty-one operations, or 19·3 per cent.; while, out of seven patients operated upon by abdominal hysterectomy for fibrous tumour, no less than six died, or a mortality of 85·7 per cent. In other forms of operation the results were very unfortunate: but the totals were not large enough to permit a fair expression of the facts in percentages. Speaking generally, 636 patients were admitted during the year; 373 were operated upon, with 26 deaths, or close upon 7 per cent., and among the 263 not operated upon the deaths were 11, or 4·19 per cent. Of the twenty-six deaths after operation, twenty are said by the committee to have been due to septicæmia, or to have been accompanied by symptoms of septic poisoning. The committee point out that the mortality after ovariotomy was double the average, while that after hysterectomy was so great that, if sustained, it would be prohibitory of the operation. Exploratory incision was performed in nine cases. In five of them no disease was found, or no organ so diseased as to require removal. Four of the patients died, giving a mortality of 44·4 per cent. It is unfortunate that the committee does not state whether the deaths were of the persons who presented no disease or of the others. They note that septic temperatures were not uncommon among patients operated upon who ultimately recovered, and also, as a point bearing upon the general sanitary state of the hospital, that these temperatures did not prevail in the practice of every member of the staff. It is interesting also to observe in this connection that the report acknowledges having received "every assistance (with one exception) from the medical staff of the hospital."

Making due allowance for the circumstance that one of the patients who died (after the removal of a sub-mucous fibroid) is said to have been "septic on admission"), we have during the year 1893 a total of nineteen deaths from a preventible cause; and anyone conversant with hospital work, who reads between the lines of the report, will have no great difficulty in understanding how it was that these deaths were permitted to occur. The institution does not seem to have been in any proper sense a hospital, that is to say, an institution in which the members of a medical staff were working in unison for the public good, but simply a house, in a very unwholesome condition, in which three different doctors performed whatever operations they pleased, and performed these operations in any manner that seemed good to them. It appears to be probable that at least one of the physicians does not believe in the utility of antiseptic precautions, and therefore does not employ them; but one of the principal uses of a "staff" is to restrain individual eccentricities within due bounds, and to hinder them from being displayed in a manner injurious to patients. Such a history as that which we have just related would have been impossible at any general hospital, where, as a rule, a consultation between members of the staff is required and held prior to any operation which endangers life and is not of immediate urgency in point of time, and where any excess of mortality among the patients of one surgeon would speedily attract the attention of his colleagues.

Who, then, is to blame. Primarily, we think, the public, who give money to so-called medical charities without any assurance that the institutions thus supported are worthy of the names which they assume, and who will not even take the simple precaution of putting a question on the subject to some physician or surgeon who is above the suspicion of interested motive. If we look round at the too numerous small special hospitals we shall find that some of them have been established as a joint speculation, by some pushing young doctor and some pushing "secretary"; that they are frequently officered by men who would never be placed upon the staff of any great hospital, and who have no common interest or common pride in the character of the institution which they profess to serve. Each officer to such an institution fights for his own hand, and seeks to use his patients as raw material from which to work up a reputation for himself. Nothing is done in them which would not be done far better, and at less cost, in the wards of a great hospital; and precautions are habitually neglected which in the great hospital would be observed as a matter of course.

We trust that Lord Cadogan, whose public spirit and right-mindedness are well known, will feel that he is called upon to take some decided action, and that in this the bulk of the subscribers will support him. Nineteen women, according to the report, lost their lives from septicæmia in 1893, and many more were placed in jeopardy, and had "septic temperatures." There is no question here of the propriety of individual operations, but only of the absence of proper care in the general conduct of the surgical work of the institution. The "Consulting Surgeon," Mr. Jonathan Hutchinson, is a past President of the Royal College of Surgeons of England; and he, as well as the authorities of that College generally, might be inclined to suggest that the whole history throws much light upon the fitness for survival of that new result of gynæcological evolution, the operating physician; and to point out that the Fellowship of the College is possessed, according to the advertisement of the hospital in the current volume of the "Medical Directory," by only one out of the nine physicians and assistant physicians who constitute the acting staff. If the money which has been worse than wasted at Chelsea had been given, say, to St. George's, all the good done at the former place might have been accomplished, and the nineteen lives would probably have been spared. We hope to see the time when every professing "hospital" will be subjected to periodical examination by experts before it is allowed to keep open its doors for the reception of the sick; and, in the meanwhile, such an example as that furnished by Chelsea ought not to be wholly thrown away. The chief fear is lest the nature of the case should be misrepresented by ignorant people, who may use it as furnishing arguments in condemnation of hospitals generally, instead of for condemning the absence of all the more important precautions under which their work should be conducted.

